# HLA sensitisation: can it be prevented?

**DOI:** 10.1007/s00467-014-2868-6

**Published:** 2014-07-26

**Authors:** Lesley Rees, Jon Jin Kim

**Affiliations:** 1 Department of Paediatric Nephrology, Great Ormond Street Hospital for Children NHS Foundation Trust, Great Ormond Street, London, WC1N 3JH UK; 2 MRC Centre for Transplantation, Guy’s Hospital, Great Maze Pond, London, SE1 9EH UK; 3 Institute of Child Health, University College London, London, UK

**Keywords:** HLA antibodies, Sensitisation, Red blood cell transfusion, Renal transplantation, Transplant waiting times

## Abstract

Human leukocyte antigen (HLA) sensitisation occurs after transfusion of blood products and transplantation. It can also happen spontaneously through cross-sensitisation from infection and pro-inflammatory events. Patients who are highly sensitised face longer waiting times on organ allocation programmes, more graft rejection and therefore more side effects of immunosuppression, and poorer graft outcomes. In this review, we discuss these issues, along with the limitations of modern HLA detection methods, and potential ways of decreasing HLA antibody development. We do not discuss the removal of antibodies after they have developed.

## Introduction

Antibodies to human leukocyte antigens (HLA) are an important barrier to transplantation. When directed against donor HLA they can cause acute graft rejection and chronic graft nephropathy. HLA-sensitised patients may meet with difficulty and delay in finding an HLA-compatible graft, leading to longer waiting times on the transplant list. Their presence is of particular importance in children, who are likely to need more than one transplant in their lifetime.

## Human leukocyte antigens and HLA sensitisation

The major histocompatibility complex (MHC) located on chromosome 6 consists of a linked set of genetic loci containing many genes involved in the immune response, including the HLA genes. The products of these genes are expressed on the cell surface as glycoproteins, of which there are three classes within the MHC region:

- Class I region, which includes the HLA genes HLA-A, -B and -C, expressed on nearly all nucleated cells
- Class II region, which includes HLA genes HLA-DR, -DQ and -DP only expressed on B cells, antigen-presenting cells (APCs) and on activated endothelial cells (that can act as APCs)
- Class III region, which includes the genes for components of the complement cascade and cytokines, e.g. TNF, LTA

Antigen-presenting cells (APCs) are a group of cells that process antigens and present them, in association with HLA molecules, to T cells. CD4 T cells (T helper cells) interact with class II molecules, resulting in the production of cytokines that lead to a cascade of cellular and humoral reactions that are responsible for the effector responses important in transplant rejection. CD8 T cells (T killer cells) are cytolytic, directly interacting with cells expressing class I and maybe toxic to the cell to which they bind.

Human leukocyte antigen antibodies can develop under any circumstance of exposure to non-self HLA antigens. They may be unique to a specific allele or limited group or recognise an epitope that is shared by more than one HLA molecule resulting in cross-reactivity. The level of sensitisation (called reaction frequency [RF]) for a patient is calculated by finding the percentage of blood group identical, HLA-incompatible donors in the donor pool: i.e. if the patient’s serum reacts with 50 % of a panel of sera that is representative of the donor pool, then half of donors would be expected to give a positive cross-match and be unacceptable. HLA antibodies therefore represent a serious obstacle to successful transplantation. Pre-transplant identification of preformed HLA antibodies is essential in order to predict whether a potential donor will be HLA compatible and to avoid unnecessary consideration of an inappropriate donor [1].

## Modern methods of HLA antibody measurement: are we measuring what we think we are?

**Fig. 1:**
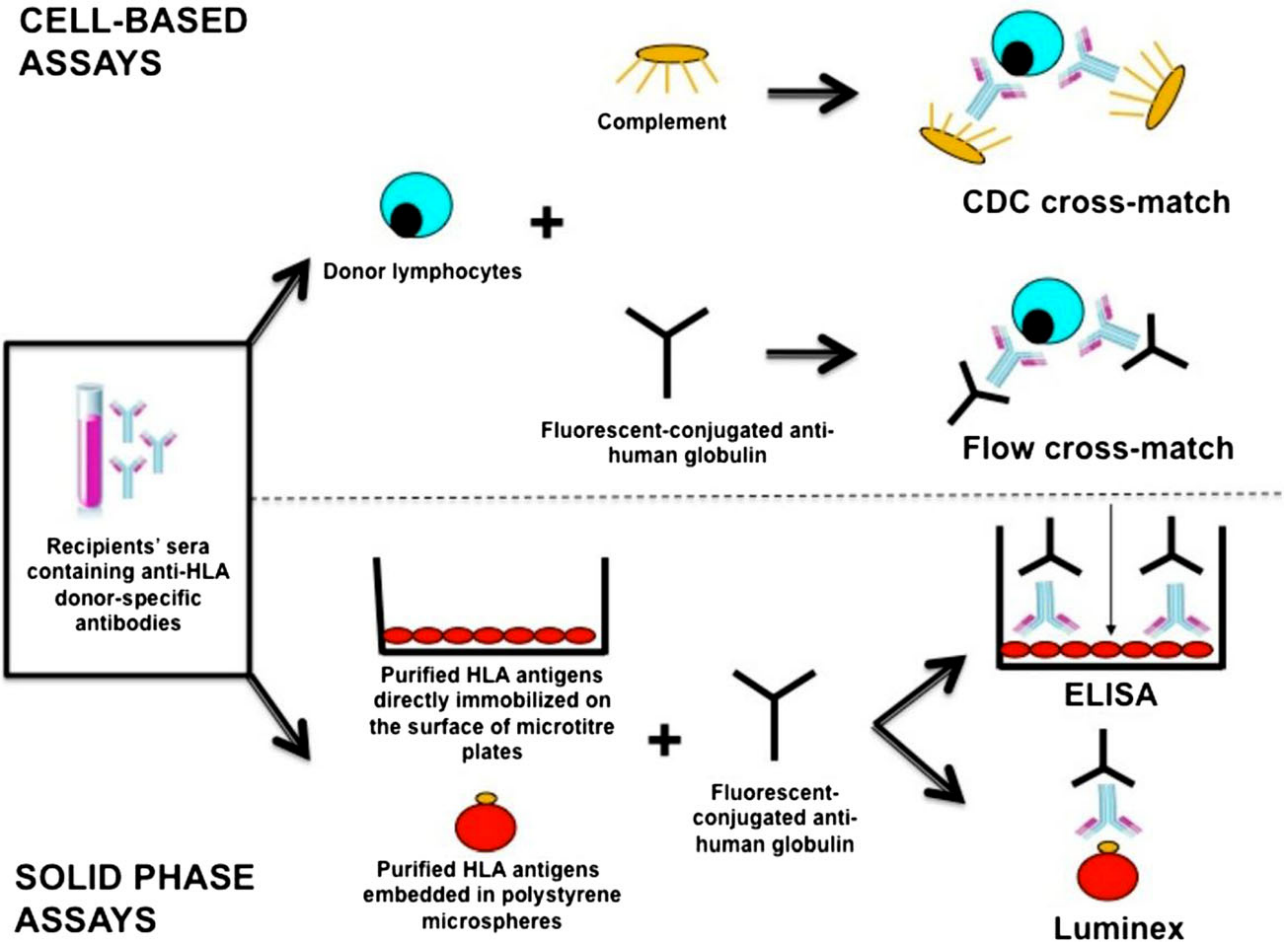
Methods of human leukocyte antigen (HLA) antibody detection. Adapted from Dheda et al. [4], originally published under CC BY 3.0 license

Historically, the detection of HLA antibodies was based on complement-dependent cytotoxicity (CDC), where sera were incubated with a panel of cells with the addition of complement and the read out was cell lysis (Fig. 1) [2–5]. Sensitivity for detecting antibodies is low, but the positive predictive value for early antibody mediated rejection is high. The sensitivity can be improved by using flow cytometry to detect the bound antibodies. By concurrently staining for T-cells and B-cells HLA class I and HLA class II respectively can be typed [2].

Currently, HLA antibody screening is carried out on solid phase assays either using HLA molecules bound to plates in an ELISA system or more commonly polystyrene beads using the Luminex platform [2, 3] (Fig. 1). Each bead is coated with a single cloned recombinant HLA epitope. With the large library of HLA alleles available, this has allowed for detection of HLA antibodies across all 11 HLA loci, including rare alleles in the population and the evaluation of complexly sensitised sera down to the level of individual specificities [2]. The ability to routinely test for HLA antibodies against HLA-Cw, -DQA, -DQB, -DPA, and -DPB has also lead to a greater appreciation of their role in chronic antibody-mediated rejection post-transplant [2, 6].

The quantity of antibody is measured by the mean fluorescence intensity (MFI) of each bead corresponding to the level of antibody bound. As there is large variability between labs and even between tests done by the same lab, the MFI measurement is only semi-quantitative [2]. This inherent variability is due to the sensitive nature of the assay but can also be due to differences between densities of the antigens on each bead from different manufacturers. Even in the setting of a study using a fixed protocol, variations of 25 % were found [7]. In addition, antibodies to common HLA epitopes can give artificially low MFI values if they bind to multiple beads sharing similar epitopes, giving a large breadth of antigen positivity but low MFI values [2]. The constructed recombinant HLA allele on beads may not directly correspond to the three-dimensional HLA structure in vivo on cell surfaces and might not represent exposed epitopes [8]. In addition, false-positive results can be obtained by binding of antibody to denatured protein on the beads [9].

At a practical level, most laboratories use a combination of cell based and solid phase assays. Pre-transplantation sera are screened using HLA Class I and II panels. Positive results are often confirmed using kits from separate manufacturers before proceeding to specific HLA antibody typing using solid phase assays. There are no fixed standard MFI thresholds that predict a positive CDC crossmatch and represent a contra-indication to transplantation [2]. Interpretation of MFI values must be made according to local guidelines in close collaboration with HLA laboratories and take into account potential past significant sensitisation events. Of note, certain therapies, particularly immunoglobulin-based therapies such as pooled IVIG, ATG, and rituximab will interfere with solid phase assays [2]. The correct interpretation of HLA antibody screening is important to avoid disadvantaging recipients with positive results that might not be clinically significant.

## Causes of HLA sensitisation

Human leukocyte antigen antibodies usually develop in association with exposure to non-self HLA molecules such as blood products, foreign tissue during transplantation or during pregnancy, but they can also develop spontaneously.

### Blood products

Despite the availability of erythropoiesis-stimulating agents (ESAs), anaemia remains a significant problem worldwide. An analysis from the International Pediatric Peritoneal Dialysis Network has shown that of 1,394 paediatric patients undergoing peritoneal dialysis from 30 countries, 25 % of patients had haemoglobin levels below target (<10 g/dl or <9.5 g/dl in children older or younger than 2 years respectively) despite the prescription of ESAs to 92 % of patients [10]. These children are vulnerable to developing a blood requirement during acute illness; thus, optimisation of ESA therapy may at least be able to prevent blood transfusion in these patients. However, there remain situations when blood transfusions cannot be avoided: for example, infants on haemodialysis who need lines priming with blood; the child with late presentation in chronic kidney disease (CKD) stage 5; in emergencies; or during surgical procedures.

The sensitisation risk from red cell transfusions is often underestimated owing to the popular misconception that erythrocytes (being enucleated cells) do not express HLA molecules. In actual fact, erythrocytes express low levels of HLA class I molecules, i.e. 100–2,000 per cell (compared to 1−2 × 10^5^ on leukocytes) [11, 12]. However, because of the large numbers of erythrocytes, HLA sensitisation would be expected to occur. Several studies in the adult literature using contemporary solid phase assay methods have confirmed that blood transfusions induce or reactivate HLA allo-immunisation [13–16]. The risk of sensitisation is proportional to prior sensitisation events. Therefore, children with failed transplants are at the highest risk of sensitisation, followed by those who have received multiple transfusions [11, 17]. In a study of male patients awaiting their first transplants, transfusion with leukodepleted red blood cells was associated with a relative risk of 4.1 of developing HLA antibodies compared with non-transfused patients [15]. In another study of patients on dialysis, transfused patients had an adjusted odds ratio of 9.6, even when applying a high positive criterion of MFI >10,000. The risk remained even when patients with pro-inflammatory events were excluded [16]. The risk of sensitisation in paediatrics is less well characterised, although it is reported that children are more likely to mount an alloimmune response than adults [18]. Infants are a special group as they are less likely to develop allo-sensitisation, even though they need blood priming of haemodialysis lines [19]. In our centre, 1 in 7 (16 %) developed HLA antibodies despite an average of 7.5 adult units per individual [19]. Some HLA antigens may be more immunogenic than others, for example, HLA-A3, A66, and B18 [20, 21]. In summary, red cells pose a significant risk of HLA sensitisation, which is stratified according to prior sensitisation events.

Other blood products that contain leukocytes or platelets are also immunogenic, and are often needed alongside blood in the sick child. Platelets in particular contain 81,587 (±20,016) HLA molecules per cell [22]. Platelet refractoriness is a recognised complication of multiple platelet transfusions in the setting of malignancies and often requires treatment with HLA-matched platelets. The investigation of HLA sensitisation in CKD patients who do not have such high platelet requirements has not been published. Leukocytes, white cell fragments, DNA, HLA peptides, cell debris and pro-inflammatory cytokines that accumulate in the fluid suspension of the blood product during storage can all potentially induce sensitisation with transfusion [23, 24].

### Transplantation

Transplant programmes for children with CKD have been very successful, with more children undergoing transplantation, and at progressively younger ages. Indeed, transplantation is generally considered to be the optimal management for children needing renal replacement therapy (RRT). However, despite improvements in outcomes over the decades, some children will inevitably lose their transplants and require relisting for repeat transplantation. Many of these children will develop HLA antibodies, although antibodies do not inevitably develop to every mismatched antigen [25]. Even if patients are HLA-antibody-negative at the time of graft loss, a proportion can subsequently become positive, usually owing to cessation of immunosuppression or a triggering pro-inflammatory event, including a blood transfusion [11, 26]. There has been a report of two patients who were initially unsensitised, then developed HLA antibodies to the previous transplant after transfusion but not towards the blood donor [27].

Another group of children that is increasing in size and complexity consists of those who have received transplants for other organ failure. These patients often have renal dysfunction even before transplantation owing to co-morbidity from their underlying condition and develop further nephrotoxicity related to calcineurin inhibitor therapy [28]. The prevalence of HLA antibodies in these patients is high and varies from 12 to 92 % depending on organ type and study [29]. HLA matching is often not performed in other solid organ transplants as other factors often take priority, such as size/weight and the lack of time to perform cross-matching in order to reduce ischaemia–reperfusion injury [29, 30]. In addition, cardiac patients have high HLA sensitisation levels owing to large transfusion requirements peri-operatively and the use of ventricular assist devices has also been linked to HLA sensitisation [11]. In summary, transplantation of any solid organs represent the largest sensitisation risk because of the large antigen load. Efforts at prolonging allograft survival will have a direct impact on HLA sensitisation and the need for retransplantation.

### Spontaneous development of HLA antibodies

Human leukocyte antigen antibodies have been detected in patients without any known sensitisation events, and are called “spontaneous” HLA antibodies. In a study of a paediatric population of 23 children awaiting transplant who did not receive blood products 26 % developed HLA antibodies over a course of 19 months [19]. A recent large cross-sectional study of UK adult male patients awaiting their first transplants showed a sensitisation rate of 4.1 % [13, 20]. This could be due to cross-sensitisation following infections, such as influenza and hepatitis C [31–33]. Environmental factors such as microorganisms, ingested proteins, and allergens have also been proposed as potential causes of the variation in “spontaneous” HLA antibody detection rates, and allosensitisation rates as high as 64 % have been detected in healthy adult Mexican blood donating males [34]. Other pro-inflammatory events such as operations and trauma have also been associated with an increase in HLA antibodies [35]. There has also been concerns regarding the potential for HLA allosensitisation following vaccinations [33]. However, as these studies were carried out using solid phase assays, the clinical significance of the positive results is not well established. In fact, the majority of the HLA antibodies declined or disappeared on follow-up testing [11, 33]. This was also the case in the study examining HLA sensitisation following H1N1 influenza vaccinations and patients could also be allosensitised following actual influenza infection as it was highly prevalent at the time of the study [33]. Other studies examining HLA sensitisation after immunisation have not shown an adverse effect [36, 37]. Therefore, established vaccinations remain recommended in children with CKD.

In summary, “spontaneous” HLA antibodies are likely non-specific and arise from an upregulation of the immune system from pro-inflammatory events. This is different from the setting post-transplant, where infection is recognised to trigger rejection episodes [38].

## HLA sensitisation and organ allocation algorithms worldwide

The predominant factor determining the incidence of HLA sensitisation in any population is the transplant HLA matching policy, which varies around the world and even within the same country: for example, in Belgium and Holland, HLA matching schemes vary from acceptance of all mismatches to a minimal match of 1A, 1B, and 1DR [39]. In the UK, the aim of the allocation programme is to maximise outcomes and minimise resultant sensitisation by the prioritisation of well-matched transplants for younger patients [25]. In the USA the transplant allocation policy favours reducing time on dialysis over HLA matching [40].

Sensitisation increases in proportion to the number of HLA mismatches that a patient is exposed to. It can occur even with a zero mismatched graft, although only in up to 10 % of patients in this group [5]. In the USA, up to 30 % of all patients waiting for a renal transplant have a reaction frequency that is greater than 20 % [41]. HLA sensitisation by blood is also important. The United States Renal Data System (USRDS) 2010 report has shown that patients who have received transfusions have a 2.38 greater odds of a reaction frequency >80 %. It would appear that the breadth and quantity of HLA antibody development is the most severe in those who are already sensitised [42].

## Why is HLA matching important?

### Effect on graft outcome

**Fig. 2:**
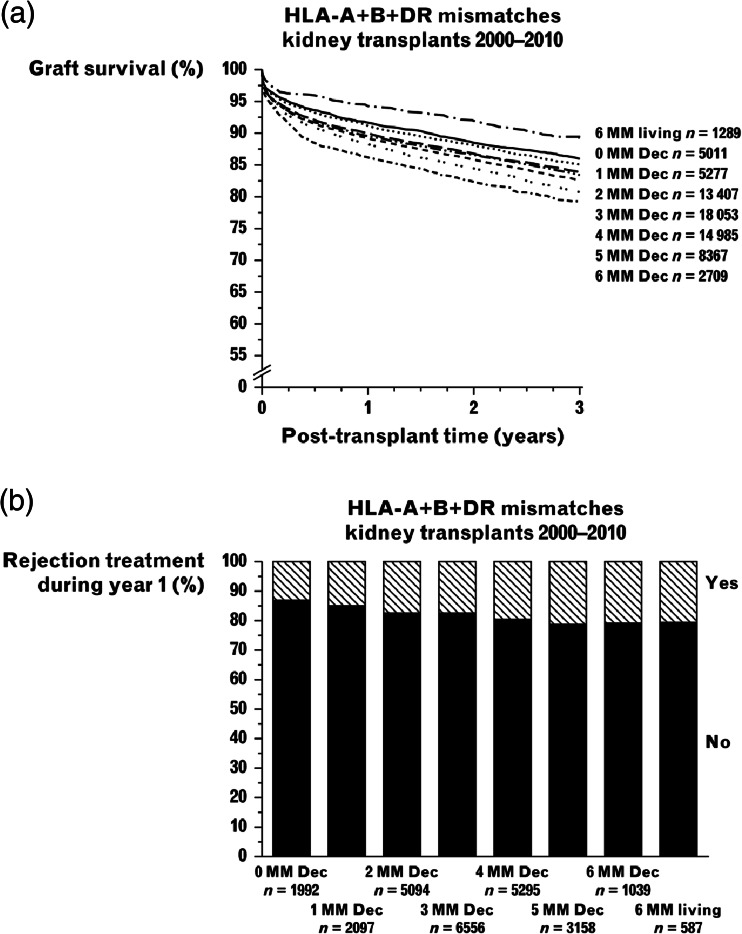
**a** Graft survival according to human leukocyte antigens (HLA) mismatches. **b** Incidence of rejection according to HLA mismatches. Data for recipients of deceased donor transplants and poorly matched living donors reported to the Collaborative Transplant Study, 2000–2010. Used with permission from Susal and Opelz [5]

It has been argued that an HLA-matching policy extends time on dialysis, with all its well-known adverse effects; that the benefits of matching may be outweighed by prolongation of the cold ischaemia time in organ sharing schemes that cover large geographical areas; and that with the success of current immunosuppression, HLA matching is not necessary [43]. Indeed, donor and recipient matching at the HLA-A and HLA-B locus has been excluded in the USA from the kidney allocation scheme because the benefits to survival were shown to be small and to increase waiting times [44]. An analysis of all first deceased donor transplants from donors less than 35 years of age in 1,585 children between 1996 and 2004 in the USA has suggested that this should apply to HLA-DR matching as well. This study, which excluded zero HLA-mismatched kidneys, showed 5-year graft survival of 71 % for no mismatches at HLA-DR, 69 % for one mismatch, and 71 % for two mismatches. The conclusion from this review was that paediatric recipients should be prioritised for such kidneys and HLA-DR mismatch should be discounted [43]. However, this study is in contradiction to others, including paediatric studies, which have shown that HLA matching is one of the most important influences on outcome. Furthermore, the study did not look at rejection rates or immunosuppression requirement and its adverse effects [45]. More recently, the benefits of HLA matching have been very clearly shown in a much larger adult cohort reported to the Collaborative Transplant Study. For every increase in mismatch there was a decrease in graft survival (Fig. 2a). This matching effect also extends to living donation, with a 92 % transplant survival at 5 years for an HLA-identical sibling and 86 % for one-haplotype donors, compared with 76 % for deceased donors [5].

### Rejection episodes

Much of the poorer outcome of mismatched grafts can be explained by an increased rate of rejection episodes, which rises in proportion to the number of mismatches (Fig. 2b). In turn, the greater immunosuppression requirement is associated with an increase in complications, including osteoporosis, hip fractures and death from infection in adult studies, and non-Hodgkin lymphoma [5]. The latter has been shown to be two-fold more common with two HLA-DR mismatches in a study of 9,000 children [46].

### Donor-specific HLA antibody development

Approximately two thirds of late graft loss is due to antibody-mediated rejection. Fifteen to twenty per cent of patients develop de novo donor-specific HLA antibodies (DSA) by 5 years post-transplant and 50 % of these patients subsequently lose their grafts within the next 5 years. The primary risk of developing DSA is the degree of HLA mismatch, particularly at HLA-DR and -DQ [5, 47, 48]. The risk is compounded by a reduction of immunosuppression (either in immunosuppression minimisation protocols or due to complications such as infections) and by non-adherence [49, 50]. Patients with DSA towards HLA-DQ are at an increased risk of developing transplant glomerulopathy, which is often difficult to treat and progresses to loss of the renal allograft [6, 51]. In our cohort at Great Ormond Street Hospital, 45 % of DSA-positive patients had HLA-DQ antibodies (Jon J. Kim, personal communication, May 2014). HLA-DQ matching is currently not being performed in renal transplantation but is a consideration for the future.

Studies looking at the prevention of DSA have focused on patients receiving blood-group incompatible transplantation (ABOi) as these patients undergo B-cell depletion with either splenectomy (historically) or rituximab. The hypothesis is whether B-cell depletion might remove memory B-cells and have an immune-educating effect on emerging B-cells after transplantation. The results from two retrospective cohorts are conflicting with one showing a reduction in the formation of de novo DSA and one showing no effect [52, 53]. However, interim results at 3 years′ follow-up of a randomised control trial (RCT) of rituximab at induction showed an increasing trend for DSA formation and a significant increase in mortality owing to cardiac arrests in the rituximab group [54]. Another RCT had to be aborted early owing to increased rates of rejection in the rituximab group in the setting of steroid avoidance [55]. These studies highlight that non-specific depletion of B-cells can be detrimental as it also depletes the subpopulation of B-cells, which has recently been shown to play a regulatory role [56]. A third RCT is underway (ReMIND) [57]. In summary, avoidance of HLA mismatching and judicious immunosuppression levels are still the best strategies for the prevention of de novo DSA formation.

## Effects of preformed HLA antibodies

An analysis of the US Scientific Registry of Transplant Recipients, which studied 2,704 children relisted for transplant after a failed graft between 1990 and 2008, found that DR mismatching at the first transplant resulted in more sensitisation, a reduced chance of retransplantation with longer waiting times if retransplant was achieved, and poorer transplant outcome [40].

### Waiting times

The waiting time for a transplant for HLA-sensitised patients is up to four times longer than that for non-sensitised patients [41]. As would be expected, this is influenced by the breadth of HLA sensitisation, as reflected in the reaction frequency. In the USA, prioritisation of kidneys from donors <35 years of age for children has sacrificed an increase in HLA sensitisation for shorter waiting times. The US Scientific Registry of Transplant Recipients has shown that the reaction frequency overall increased from a mean of 7 to 55 % after first graft failure. This figure was 39 % in those who could be retransplanted, with a waiting time of 19 months, but was 68 % in those who failed to obtain a second transplant during the study period of 44 months. Two prior HLA-DR mismatches significantly increased the waiting time, even in those who did receive a retransplant (23 months vs 19 months), and 45 % of these patients remained un-transplanted compared with 29 % in patients with 0–1 DR mismatches [40].

### Transplant survival

**Fig. 3:**
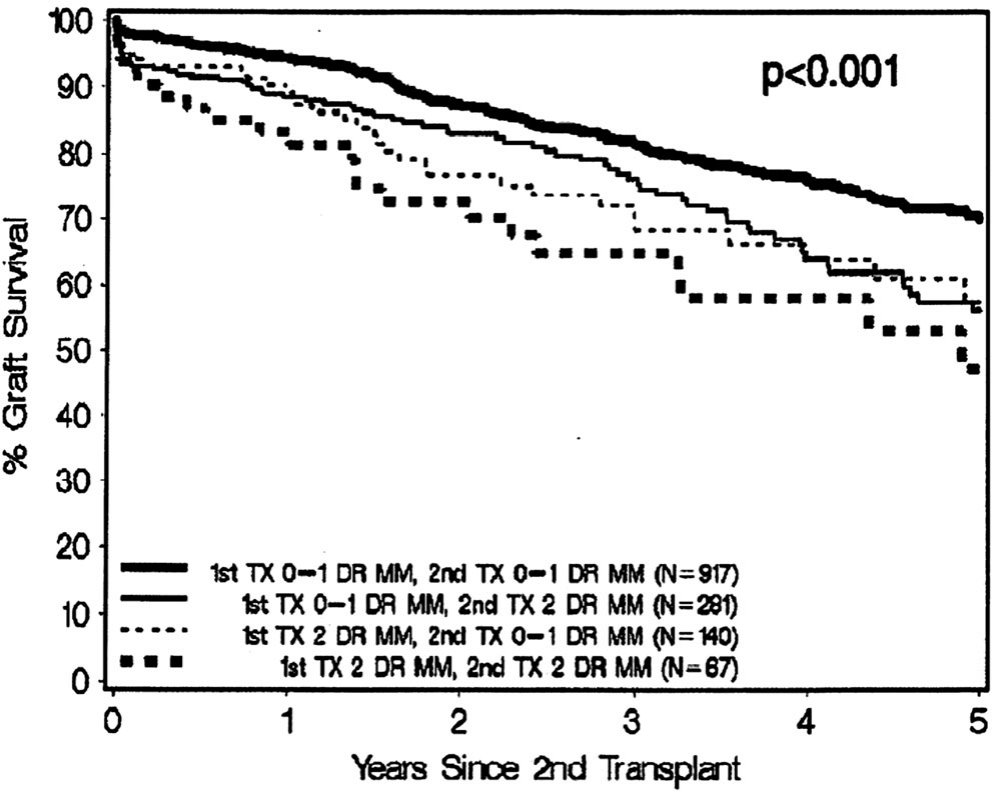
Graft survival for re-transplantations between 2000 and 2008 segregated by DR mismatches at first and second transplant. Used with permission from Gralla et al. [40]

In an analysis from The US Scientific Registry of Transplant Recipients of children receiving their second transplant between 2000 and 2008, those with 2 DR mismatches at first and second transplant had 5-year graft survival of 47 %, whereas those with 0–1 DR mismatch had the best at 70 % (Fig. 3). Although sensitisation had a significant effect, the strongest association with poor transplant outcome was two DR mismatches at both transplants [40]. A further paediatric study has shown an increased risk of rejection rates, sensitisation and transplant outcome with DR mismatch [58] and the risk of sensitisation seems to be greater in younger patients [59].

The presence of DSA pre-transplant is associated with poorer allograft outcome. In a meta-analysis of 1,119 adult patients, the presence of DSA despite a negative flow cytometry and CDC cross-match doubled the risk of antibody-mediated rejection (ABMR) and increased the risk of allograft failure (relative risk 1.76, confidence interval 1.13–2.74) [60]. In a recent study looking specifically at “spontaneous” DSA, DSA-positive patients also had higher incidences of ABMR [61]. However, these episodes responded to treatment and graft function at 1-year post-transplant was similar. Therefore, although pre-existing DSAs with negative cross-match are not a contra-indication to transplantation, patients require close monitoring for ABMR. Strategies used to desensitise patients include combinations of IVIG, plasmapheresis, rituximab and bortezomib [62].

## Prevention of HLA sensitisation

### Preventing sensitisation from transfusions

The main modifiable risk factor to reduce sensitisation is the avoidance of blood transfusions. This will require optimisation of erythropoietin and iron supplementation therapies. Where unavoidable, transfusions should only be used in emergencies or for symptomatic treatment rather than using arbitrary haemoglobin cut-offs for treatment. Close co-operation is also required from all health care professionals looking after children with CKD, including surgeons, anaesthetists and intensivists.

In the UK, universal leukodepletion was adopted in 1999. Leukocytes are filtered and residual counts of <1 × 10^6^ per unit are often achieved. The effect of leukodepletion on HLA sensitisation is controversial and studies have shown similar rates before and after universal adoption of leukodepleted blood [24, 42, 63]. In an older study randomising patients to leukodepleted red cells or non-reduced packed red cells, there was no benefit to HLA sensitisation, the likelihood of receiving a graft or graft survival [64]. It is clear, though, that the risk of HLA sensitisation is not entirely negated by leukodepletion. In the past, short courses of immunosuppression were used in the setting of a donor-specific transfusion protocol and this was associated with lower rates of HLA sensitisation, although this practice is not routinely performed currently [65, 66]. Additionally, we performed a study looking at the washing of leukodepleted blood. We have shown in vitro that the amount of residual leukocytes remains similar when using a manual wash, and there is only a 33 % reduction when using an automated method. The use of washed cells in patients has not reduced sensitisation risk either [19].

As HLA matching is performed for platelet-refractory patients, this practice can in theory be extended to red blood cells. In a small study, 37 patients were given HLA-A- and -B-matched blood where possible, and the HLAMatchmaker programme was used to allocate blood units from donors considered least likely to stimulate an immune response [67]. Development of HLA antibodies was compared with a group of 31 patients who received randomly selected blood transfusions. In the HLA-selected group, only 27 % received 0-HLA-A- and -B-mismatched blood and the rest were allocated by HLAMatchmaker, identifying the difficulties in obtaining well-matched blood even just at the class 1 level. However, none of the patients who received HLA-selected blood experienced any changes in antibody levels, whereas 7 of those given randomly selected blood had de novo HLA antibodies or an increase in panel reactive HLA antibodies [67]. This study can be interpreted as an effect of HLA matching or the usefulness of allocating with HLAMatchmaker but would obviously need a further randomised controlled trial to determine efficacy.

Even blood given perioperatively can increase ABMR in patients with preformed HLA antibodies, suggesting that transfusion might provide additional allostimulation, despite concomitant immune suppression [68].

### Avoidance of HLA mismatches with a high frequency in the donor population

The most common HLA antigen is HLA-A2, which is found in almost 50 % of Caucasian people. Ninety per cent of Caucasians with HLA-A2 have HLA-A*02:01, although other ethnic groups may have different alleles. Frequencies of other HLA antigens vary with ethnicity: e.g. HLA-B8 is present in 30 % of Irish; HLA-B54 is unique to Far Eastern populations such as Japan; and HLA-A36 is found in the Black (Afro-Caribbean) population. It may be considered unwise to mismatch at a common antigen, e.g. HLA-A2, particularly in children, to avoid sensitisation to half the potential donors, which would compromise retransplantation. Bw6 is an epitope shared by many HLA-B antigens. Antibody specificity against the Bw6 epitope is particularly problematic, as it is found in 86 % of the general population [1, 41].

In the UK, highly sensitised patients (>85 % calculated reaction frequency) are prioritised for 000 HLA-A-, -B-, -DR-mismatched grafts in order to reduce waiting times and prolong transplant survival [25].

The HLAMatchmaker mentioned above is a program undergoing development that aims to match HLA at the three-dimensional structural level and examines epitopes that are exposed on the surface of HLA. This could potentially be used to further refine HLA mismatches to low or high immunogenic risk or assign acceptable mismatches [69, 70].

### Role of weaning of immunosuppression after a failed transplant

The contribution of nephrectomy and/or continuing immunosuppression to HLA sensitisation in the failing graft is not clear. Transplants that fail in the first year, or those that result from acute non-concordance with medications often present with acute rejection and systemic involvement, and frequently progress to nephrectomy; it is perhaps not surprising, therefore, that HLA sensitisation is common in this situation [71]. Any possible benefits of HLA sensitisation by continuation of immunosuppression with a failing graft have to be balanced against the side effects and, in particular, the risk of infection in the patient on dialysis. One adult study has demonstrated the importance of continuing immunosuppression. Of 119 adult patients with a low reaction frequency before transplantation, 56 % of patients were highly sensitised (class I or II reaction frequency ≥80 %) at follow-up 6 to 24 months after transplant failure. The percentage of those who were highly sensitised increased from 21 % at the time of failure on immunosuppressive therapy to 68 % after weaning, whereas patients who maintained immunosuppression showed minimal sensitisation after failure. Transplant nephrectomy was required in 41 % of patients who were weaned from immunosuppression, but in none of those who continued immunosuppression [72]. However, this is at the cost of an increased risk of infection. Of 186 patients with failed transplants, 38 % who had been weaned from their immunosuppression had infection requiring hospitalisation, in contrast to 88 % who remained on it. Infections were predominantly dialysis catheter-related. On the other hand there was a higher incidence of graft nephrectomy in the patients who had weaned their immunosuppression (81 % vs 30 %) owing to the development of transplant rejection [73].

The practice of performing nephrectomies for failed transplants varies from centre to centre. The removal of transplants owing to early failure (<6–12 months) has been associated with a reduction in HLA sensitisation and improvement in subsequent transplant outcomes [74, 75]. In contrast, removal of late failure grafts is associated with an increase in detectable HLA antibodies [74, 76–78]. The detection of DSA increases to as high as 81–87 % post-nephrectomy using solid phase arrays [76, 78]. There are concerns that the failed allograft may serve as a reservoir absorbing circulating DSA and preventing their detection, hence the exclusion of previous mismatches from future transplants [78]. The breadth of HLA reactivity also increases and in one study, the newly detected HLA antibodies showed 89 % homology to DSA [74, 76]. The impact of HLA sensitisation notwithstanding, the decision to perform nephrectomy needs to balance the risks (bleeding, sepsis) against clinical indications (tenderness and fever, or to create space for future transplants). Preservation of residual renal function on dialysis is clearly associated with improved cardiovascular outcomes [71]. However, the retention of a failed allograft is also associated with a chronic inflammatory state that can lead to anaemia, erythropoietin resistance and hypoalbuminaemia [79]. Studies investigating outcomes on subsequent transplants have not shown any conclusive differences between nephrectomised and non-nephrectomised patients [75, 80, 81]. There is, however, a trend towards increased acute rejection and poorer graft survival in some studies but not in others, likely because of counter-action from increased immunosuppression [80, 82]. In summary, the decision to remove failed allografts needs to be done on an individual basis and should only be considered for cases with clear indications. Whilst the allograft remains in situ, the continuation of low-dose immunosuppression is associated with reduced HLA sensitisation.

## Conclusion

Human leukocyte antigen mismatching remains the main barrier to long-term allograft survival. In an ideal world, organ allocation programs would take a long-term view to reduce mismatching in children as they will require more than one transplant during their lifetime. HLA-DR mismatching in particular has been shown to reduce graft outcome in subsequent transplants. Avoidance of transfusions through the optimisation of erythropoietin and iron supplementation remains the key strategy as the risk of HLA sensitisation, even from leukodepleted red cells, remains a significant risk. The use of HLA-matched red cells deserves further studies. In patients with failed allografts, maintenance of immunosuppression and avoiding nephrectomy is associated with less HLA sensitisation, although this has to be balanced against the risks of infections and a potential chronic inflammatory state. The development of successful desensitisation programmes may help to mitigate the problem of HLA sensitisation in the future.

## Multiple choice questions (answers are provided following the reference list)

1. Which of the following is true regarding HLA?
  - Red blood cells do not possess HLA.
  - HLA class II is mainly present on antigen-presenting cells.
  - An example of HLA class II is HLA-G.
  - CD4 T-cells interact with HLA class I to cause cell lysis.
2. Which of the following patients has the highest risk of sensitisation?
  - A 6-month-old receiving red cells to prime haemodialysis lines.
  - A 2-year-old receiving a red blood cell transfusion at the time of transplantation surgery.
  - A 10-year-old girl on haemodialysis receiving platelets prior to removal of her infected line.
  - A 10-year-old girl with a failed transplant receiving a red blood cell transfusion following graft nephrectomy.
3. Which of the following is true regarding HLA-matching programs for transplantation?
  - The risk of rejection from HLA-A- and -B-mismatching is negligible.
  - Patients sensitised to HLA-DR, for example from previous -DR mismatch in a failed allograft, face significantly longer waiting times and poorer graft outcomes.
  - HLA matching is not clinically significant in the modern immunosuppressive era with the use of B-cell-depleting agents such as ATG and rituximab.
  - The presence of DSA pre-transplant is not associated with increased risks of antibody-mediated rejection.
4. What is the most effective practice for preventing HLA sensitisation?
  - Avoidance of transfusions and optimisation of erythropoiesis-stimulating agents and iron stores.
  - Using washed red cells for transfusion.
  - Using leukodepleted red cells for transfusion.
  - Using HLA-matched red cells for transfusion.
5. Regarding the management of failed allografts, which of the following is true?
  - Allograft nephrectomies should be routinely performed as the graft is no longer functioning.
  - Cessation of immunosuppression in the presence of a failed allograft in situ is not associated with sensitisation.
  - Nephrectomies are indicated for persisting anaemia despite optimisation of medical treatment.
  - Nephrectomies should not be performed as they are liable to absorb antibodies and have been found to benefit subsequent retransplantation.
